# Aqua­[*N*-phenyl-2-(quinolin-8-yl­oxy)acetamide]dinitratozinc(II)

**DOI:** 10.1107/S160053681000471X

**Published:** 2010-02-13

**Authors:** Wei-Na Wu, Yuan Wang, Ai-Yun Zhang, Rui-Qi Zhao, Qiu-Fen Wang

**Affiliations:** a Department of Physics and Chemistry, Henan Polytechnic University, Jiaozuo 454000, People’s Republic of China

## Abstract

In the title complex, [Zn(NO_3_)_2_(C_17_H_14_N_2_O_2_)(H_2_O)], the six-coordinated Zn atom is in a distorted octa­hedral geometry, the donor centers being two O atoms and one N atom from the tridentate organic ligand, a water O atom and two O atoms from two monodentate nitrate ions. In the crystal, O—H⋯O hydrogen bonds between the coordinated water mol­ecules and nitrate O atoms and N—H⋯O hydrogen bonds between the main ligand and nitrate O atoms consolidate the three-dimensional network.

## Related literature

For the synthesis of *N*-phenyl-2-(quinolin-8-yl­oxy)acetamide, see: Li *et al.* (2005 ▶); Wu *et al.* (2006 ▶). For the crystal structure of the hydrate of this mol­ecule, see: Li *et al.* (2005 ▶). For the coordination ability of related amides to lanthanides, see: Cai & Tan (2002 ▶); Wu *et al.* (2006 ▶).
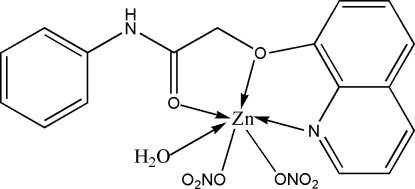


## Experimental

### 

#### Crystal data

[Zn(NO_3_)_2_(C_17_H_14_N_2_O_2_)(H_2_O)]
*M* *_r_* = 485.71
Triclinic, 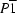

*a* = 7.9980 (14) Å
*b* = 9.5109 (16) Å
*c* = 13.359 (2) Å
α = 94.876 (2)°
β = 96.496 (2)°
γ = 106.031 (2)°
*V* = 963.2 (3) Å^3^
*Z* = 2
Mo *K*α radiation
μ = 1.34 mm^−1^
*T* = 296 K
0.30 × 0.19 × 0.11 mm

#### Data collection

Bruker SMART CCD diffractometer
Absorption correction: multi-scan (*SADABS*; Bruker, 1997 ▶) *T*_min_ = 0.745, *T*_max_ = 0.863
10541 measured reflections
4030 independent reflections
3454 reflections with *I* > 2σ(*I*)
*R*_int_ = 0.028

#### Refinement

*R*[*F* ^2^ > 2σ(*F* ^2^)] = 0.034
*wR*(*F* ^2^) = 0.093
*S* = 1.05
4030 reflections
281 parameters
H-atom parameters constrained
Δρ_max_ = 0.66 e Å^−3^
Δρ_min_ = −0.39 e Å^−3^

### 

Data collection: *SMART* (Bruker, 1997 ▶); cell refinement: *SAINT* (Bruker, 1997 ▶); data reduction: *SAINT*; program(s) used to solve structure: *SHELXS97* (Sheldrick, 2008 ▶); program(s) used to refine structure: *SHELXL97* (Sheldrick, 2008 ▶); molecular graphics: *SHELXTL* (Sheldrick, 2008 ▶); software used to prepare material for publication: *SHELXTL*.

## Supplementary Material

Crystal structure: contains datablocks I, New_Global_Publ_Block. DOI: 10.1107/S160053681000471X/bh2270sup1.cif

Structure factors: contains datablocks I. DOI: 10.1107/S160053681000471X/bh2270Isup2.hkl

Additional supplementary materials:  crystallographic information; 3D view; checkCIF report

## Figures and Tables

**Table:** 

Zn1—O6	2.0231 (17)
Zn1—O3	2.0494 (17)
Zn1—O9	2.0818 (17)
Zn1—O2	2.1110 (15)
Zn1—N1	2.1166 (17)
Zn1—O1	2.2612 (16)

**Table:** 

O6—Zn1—O3	103.05 (7)
O6—Zn1—O9	86.57 (8)
O3—Zn1—O9	170.28 (7)
O6—Zn1—O2	93.07 (7)
O3—Zn1—O2	95.75 (7)
O9—Zn1—O2	85.04 (7)
O6—Zn1—N1	120.13 (8)
O3—Zn1—N1	86.60 (7)
O9—Zn1—N1	87.29 (7)
O2—Zn1—N1	145.38 (7)
O6—Zn1—O1	163.42 (7)
O3—Zn1—O1	86.10 (7)
O9—Zn1—O1	84.93 (7)
O2—Zn1—O1	72.04 (6)
N1—Zn1—O1	73.67 (6)

**Table 2:** Hydrogen-bond geometry (Å, °)

*D*—H⋯*A*	*D*—H	H⋯*A*	*D*⋯*A*	*D*—H⋯*A*
O9—H9*A*⋯O6^i^	0.82	1.99	2.803 (3)	173
O9—H9*B*⋯O4^ii^	0.88	1.97	2.797 (3)	155
N2—H2⋯O8^iii^	0.86	2.07	2.869 (3)	155

Symmetry codes: (i) 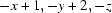; (ii) 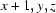; (iii) 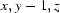.

**Fig. 1.**
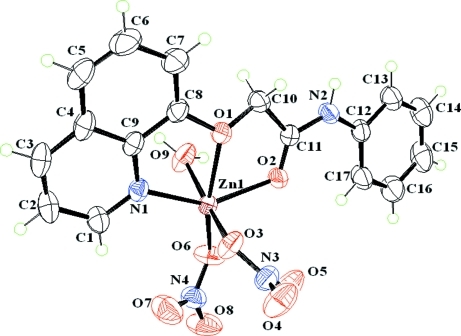
The molecular structure shown with 50% probability displacement ellipsoids.

**Fig. 2.**
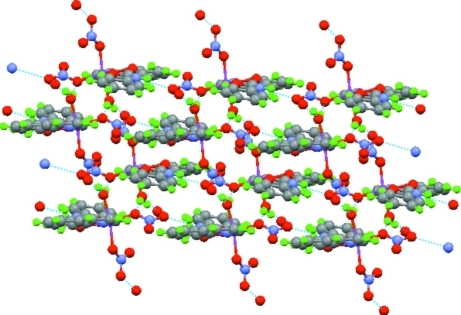
The crystal packing for the title complex via hydrogen bonds (dashed lines).

## supplementary crystallographic information

### Comment

Including our previous work (Wu *et al.*, 2006), the amide type ligands have been widely used to enhance the luminescent emissions of the lanthanide ions because of their excellent coordination ability (Cai & Tan, 2002). However, little work has been done on their transition metal complexes. Therefore, as part of our ongoing studies of the amide type ligands, the title complex was synthesized and characterized by X-ray diffraction.

As shown in Fig. 1, in the title complex, the six-coordinated Zn atom is in a distorted octahedral geometry with the donor centers of two O atoms and one N atom from the ligand, one O atom from one water molecule and two O atoms from two nitrate ions. The dihedral angle between phenyl ring and naphthyl ring is 22.5 (1)°.

In the crystal, O—H···O hydrogen bonds between the coordinated water molecules and nitrate O atoms, and N—H···O hydrogen bonds between the ligands and nitrate O atoms are helpful to consolidate the three-dimensional network (Fig. 2).

### Experimental

*N*-phenyl-2-(quinolin-8-yloxy)acetamide (Wu *et al.*, 2006; Li *et al.*, 2005) (0.278 g, 1 mmol) was dissolved in acetonitrile (10 ml), then an acetonitrile solution (10 ml) containing zinc nitrate hexahydrate (0.295 g, 1 mmol) was added dropwise at room temperature. After stirring for 2 h, the mixture was filtered and set aside to crystallize at room temperature for 8 d, giving colorless prismatic crystals.

### Refinement

Atoms H9A and H9B (water molecule O9) were located in a difference map and their positions fixed, with *U*_iso_(H) = 1.5*U*_eq_(O9). Other H atoms bonded to C and N atoms were placed in calculated positions and treated using a riding-model approximation [aromatic groups: C—H = 0.93 Å and *U*_iso_(H) = 1.2*U*_eq_(carrier C); methylene group: C—H = 0.97 Å and *U*_iso_(H) = 1.2*U*_eq_(carrier C); amine group: N—H = 0.86 Å and *U*_iso_(H2) = 1.2*U*_eq_(N2)].

### Crystal data

**Table:**

[Zn(NO_3_)_2_(C_17_H_14_N_2_O_2_)(H_2_O)]	*Z* = 2
*M**_r_* = 485.71	*F*(000) = 496
Triclinic, *P*1	*D*_x_ = 1.675 Mg m^−^^3^
Hall symbol: -P 1	Mo *K*α radiation, λ = 0.71073 Å
*a* = 7.9980 (14) Å	Cell parameters from 4639 reflections
*b* = 9.5109 (16) Å	θ = 2.6–26.4°
*c* = 13.359 (2) Å	µ = 1.34 mm^−^^1^
α = 94.876 (2)°	*T* = 296 K
β = 96.496 (2)°	Prism, colorless
γ = 106.031 (2)°	0.30 × 0.19 × 0.11 mm
*V* = 963.2 (3) Å^3^	

### Data collection

**Table:**

Bruker SMART CCD diffractometer	4030 independent reflections
Radiation source: fine-focus sealed tube	3454 reflections with *I* > 2σ(*I*)
graphite	*R*_int_ = 0.028
φ and ω scans	θ_max_ = 26.7°, θ_min_ = 2.2°
Absorption correction: multi-scan (*SADABS*; Bruker, 1997)	*h* = −10→10
*T*_min_ = 0.745, *T*_max_ = 0.863	*k* = −11→11
10541 measured reflections	*l* = −16→16

### Refinement

**Table:**

Refinement on *F*^2^	Primary atom site location: structure-invariant direct methods
Least-squares matrix: full	Secondary atom site location: difference Fourier map
*R*[*F*^2^ > 2σ(*F*^2^)] = 0.034	Hydrogen site location: inferred from neighbouring sites
*wR*(*F*^2^) = 0.093	H-atom parameters constrained
*S* = 1.05	*w* = 1/[σ^2^(*F*_o_^2^) + (0.0544*P*)^2^ + 0.1851*P*] where *P* = (*F*_o_^2^ + 2*F*_c_^2^)/3
4030 reflections	(Δ/σ)_max_ < 0.001
281 parameters	Δρ_max_ = 0.66 e Å^−^^3^
0 restraints	Δρ_min_ = −0.39 e Å^−^^3^

### Fractional atomic coordinates and isotropic or equivalent isotropic displacement parameters (Å^2^)

**Table:**

	*x*	*y*	*z*	*U*_iso_*/*U*_eq_	
Zn1	0.40610 (3)	0.96202 (3)	0.180722 (18)	0.03178 (10)	
O1	0.3965 (2)	0.74601 (17)	0.24213 (12)	0.0397 (4)	
O2	0.2694 (2)	0.78947 (17)	0.06551 (12)	0.0379 (4)	
O3	0.1934 (2)	0.9611 (2)	0.25329 (13)	0.0468 (4)	
O4	−0.0719 (3)	0.9596 (3)	0.26368 (17)	0.0721 (6)	
O5	0.0289 (3)	0.9897 (3)	0.12235 (16)	0.0820 (8)	
O6	0.4097 (3)	1.1192 (2)	0.08737 (14)	0.0524 (5)	
O7	0.3915 (4)	1.2818 (2)	0.20478 (17)	0.0757 (7)	
O8	0.3628 (4)	1.3175 (2)	0.04674 (17)	0.0769 (7)	
O9	0.6309 (2)	0.9394 (2)	0.12457 (14)	0.0566 (5)	
H9A	0.6102	0.9205	0.0626	0.085*	
H9B	0.7372	0.9403	0.1513	0.085*	
N1	0.5583 (2)	1.0166 (2)	0.32636 (13)	0.0337 (4)	
N2	0.2226 (3)	0.5535 (2)	−0.00405 (15)	0.0401 (5)	
H2	0.2306	0.4695	0.0117	0.048*	
N3	0.0477 (3)	0.9699 (2)	0.21100 (16)	0.0447 (5)	
N4	0.3863 (3)	1.2425 (2)	0.11447 (17)	0.0480 (5)	
C1	0.6333 (3)	1.1531 (3)	0.36958 (19)	0.0450 (6)	
H1	0.6103	1.2301	0.3375	0.054*	
C2	0.7469 (4)	1.1865 (3)	0.4625 (2)	0.0530 (7)	
H2A	0.7961	1.2840	0.4912	0.064*	
C3	0.7839 (4)	1.0767 (3)	0.50957 (19)	0.0528 (7)	
H3	0.8605	1.0985	0.5703	0.063*	
C4	0.7068 (3)	0.9290 (3)	0.46704 (17)	0.0431 (6)	
C5	0.7382 (4)	0.8075 (4)	0.5099 (2)	0.0566 (7)	
H5	0.8126	0.8223	0.5711	0.068*	
C6	0.6612 (4)	0.6687 (4)	0.4632 (2)	0.0608 (8)	
H6	0.6842	0.5897	0.4927	0.073*	
C7	0.5464 (4)	0.6413 (3)	0.37011 (19)	0.0510 (7)	
H7	0.4958	0.5456	0.3383	0.061*	
C8	0.5119 (3)	0.7573 (3)	0.32834 (17)	0.0370 (5)	
C9	0.5925 (3)	0.9046 (3)	0.37403 (16)	0.0343 (5)	
C10	0.3655 (3)	0.6234 (3)	0.16737 (18)	0.0411 (5)	
H10A	0.2877	0.5363	0.1880	0.049*	
H10B	0.4750	0.6033	0.1567	0.049*	
C11	0.2808 (3)	0.6650 (2)	0.07116 (17)	0.0339 (5)	
C12	0.1501 (3)	0.5535 (3)	−0.10558 (18)	0.0381 (5)	
C13	0.1263 (4)	0.4259 (3)	−0.1706 (2)	0.0494 (6)	
H13	0.1577	0.3465	−0.1465	0.059*	
C14	0.0558 (4)	0.4166 (3)	−0.2714 (2)	0.0610 (8)	
H14	0.0408	0.3311	−0.3150	0.073*	
C15	0.0081 (4)	0.5329 (3)	−0.3068 (2)	0.0629 (8)	
H15	−0.0394	0.5267	−0.3744	0.076*	
C16	0.0308 (4)	0.6595 (3)	−0.2417 (2)	0.0604 (8)	
H16	−0.0018	0.7382	−0.2661	0.072*	
C17	0.1011 (4)	0.6711 (3)	−0.1408 (2)	0.0493 (6)	
H17	0.1154	0.7566	−0.0974	0.059*	

### Atomic displacement parameters (Å^2^)

**Table:**

	*U*^11^	*U*^22^	*U*^33^	*U*^12^	*U*^13^	*U*^23^
Zn1	0.03517 (16)	0.03244 (16)	0.02720 (15)	0.01224 (11)	−0.00243 (10)	0.00149 (10)
O1	0.0471 (9)	0.0359 (9)	0.0332 (8)	0.0153 (7)	−0.0107 (7)	−0.0015 (7)
O2	0.0448 (9)	0.0303 (8)	0.0348 (8)	0.0113 (7)	−0.0075 (7)	−0.0007 (6)
O3	0.0357 (9)	0.0719 (12)	0.0367 (9)	0.0229 (8)	0.0013 (7)	0.0082 (8)
O4	0.0428 (11)	0.1124 (19)	0.0713 (15)	0.0334 (12)	0.0168 (10)	0.0182 (13)
O5	0.0611 (14)	0.148 (2)	0.0473 (13)	0.0476 (15)	−0.0026 (10)	0.0271 (14)
O6	0.0806 (13)	0.0421 (10)	0.0500 (11)	0.0342 (10)	0.0243 (10)	0.0164 (8)
O7	0.115 (2)	0.0597 (14)	0.0523 (13)	0.0335 (13)	0.0008 (12)	−0.0042 (11)
O8	0.126 (2)	0.0575 (13)	0.0664 (14)	0.0540 (14)	0.0119 (13)	0.0232 (11)
O9	0.0378 (10)	0.0931 (15)	0.0413 (10)	0.0280 (10)	0.0000 (8)	−0.0016 (10)
N1	0.0325 (10)	0.0397 (11)	0.0271 (9)	0.0100 (8)	−0.0001 (7)	0.0016 (8)
N2	0.0509 (12)	0.0290 (10)	0.0365 (11)	0.0114 (9)	−0.0060 (9)	−0.0010 (8)
N3	0.0375 (11)	0.0521 (13)	0.0450 (12)	0.0175 (9)	−0.0009 (9)	0.0022 (10)
N4	0.0587 (14)	0.0367 (11)	0.0504 (13)	0.0177 (10)	0.0046 (11)	0.0070 (10)
C1	0.0481 (14)	0.0432 (14)	0.0376 (13)	0.0074 (11)	0.0004 (11)	−0.0016 (11)
C2	0.0539 (16)	0.0532 (16)	0.0370 (14)	−0.0016 (13)	−0.0032 (12)	−0.0062 (12)
C3	0.0450 (15)	0.0700 (19)	0.0307 (13)	0.0029 (13)	−0.0070 (11)	−0.0007 (12)
C4	0.0370 (13)	0.0630 (16)	0.0257 (11)	0.0110 (11)	−0.0010 (9)	0.0032 (11)
C5	0.0592 (17)	0.079 (2)	0.0327 (13)	0.0256 (15)	−0.0087 (12)	0.0133 (14)
C6	0.077 (2)	0.068 (2)	0.0456 (16)	0.0351 (17)	−0.0035 (14)	0.0193 (14)
C7	0.0645 (17)	0.0495 (15)	0.0398 (14)	0.0212 (13)	−0.0035 (12)	0.0092 (12)
C8	0.0382 (12)	0.0444 (13)	0.0282 (11)	0.0142 (10)	−0.0012 (9)	0.0045 (9)
C9	0.0299 (11)	0.0461 (13)	0.0263 (11)	0.0110 (9)	0.0023 (8)	0.0043 (9)
C10	0.0516 (14)	0.0333 (12)	0.0362 (12)	0.0150 (10)	−0.0056 (10)	−0.0008 (10)
C11	0.0323 (11)	0.0328 (12)	0.0340 (12)	0.0080 (9)	−0.0006 (9)	0.0021 (9)
C12	0.0377 (12)	0.0352 (12)	0.0351 (12)	0.0043 (9)	−0.0016 (9)	−0.0008 (9)
C13	0.0568 (16)	0.0402 (14)	0.0447 (14)	0.0107 (12)	−0.0035 (12)	−0.0051 (11)
C14	0.072 (2)	0.0539 (17)	0.0426 (15)	0.0062 (14)	−0.0029 (14)	−0.0165 (13)
C15	0.075 (2)	0.0621 (19)	0.0345 (14)	0.0007 (15)	−0.0108 (13)	−0.0008 (13)
C16	0.073 (2)	0.0503 (17)	0.0489 (16)	0.0117 (14)	−0.0149 (14)	0.0106 (13)
C17	0.0614 (17)	0.0370 (13)	0.0413 (14)	0.0084 (12)	−0.0077 (12)	−0.0008 (11)

### Geometric parameters (Å, °)

**Table:**

Zn1—O6	2.0231 (17)	C3—C4	1.408 (4)
Zn1—O3	2.0494 (17)	C3—H3	0.9300
Zn1—O9	2.0818 (17)	C4—C5	1.403 (4)
Zn1—O2	2.1110 (15)	C4—C9	1.418 (3)
Zn1—N1	2.1166 (17)	C5—C6	1.354 (4)
Zn1—O1	2.2612 (16)	C5—H5	0.9300
O1—C8	1.369 (3)	C6—C7	1.418 (4)
O1—C10	1.416 (3)	C6—H6	0.9300
O2—C11	1.220 (3)	C7—C8	1.358 (3)
O3—N3	1.264 (3)	C7—H7	0.9300
O4—N3	1.238 (3)	C8—C9	1.421 (3)
O5—N3	1.214 (3)	C10—C11	1.518 (3)
O6—N4	1.266 (3)	C10—H10A	0.9700
O7—N4	1.225 (3)	C10—H10B	0.9700
O8—N4	1.228 (3)	C12—C17	1.384 (4)
O9—H9A	0.8200	C12—C13	1.385 (3)
O9—H9B	0.8812	C13—C14	1.385 (4)
N1—C1	1.320 (3)	C13—H13	0.9300
N1—C9	1.360 (3)	C14—C15	1.370 (4)
N2—C11	1.339 (3)	C14—H14	0.9300
N2—C12	1.414 (3)	C15—C16	1.379 (4)
N2—H2	0.8600	C15—H15	0.9300
C1—C2	1.409 (4)	C16—C17	1.384 (4)
C1—H1	0.9300	C16—H16	0.9300
C2—C3	1.347 (4)	C17—H17	0.9300
C2—H2A	0.9300		
			
O6—Zn1—O3	103.05 (7)	C5—C4—C3	124.3 (2)
O6—Zn1—O9	86.57 (8)	C5—C4—C9	119.0 (2)
O3—Zn1—O9	170.28 (7)	C3—C4—C9	116.6 (2)
O6—Zn1—O2	93.07 (7)	C6—C5—C4	120.6 (2)
O3—Zn1—O2	95.75 (7)	C6—C5—H5	119.7
O9—Zn1—O2	85.04 (7)	C4—C5—H5	119.7
O6—Zn1—N1	120.13 (8)	C5—C6—C7	121.5 (3)
O3—Zn1—N1	86.60 (7)	C5—C6—H6	119.3
O9—Zn1—N1	87.29 (7)	C7—C6—H6	119.3
O2—Zn1—N1	145.38 (7)	C8—C7—C6	118.8 (3)
O6—Zn1—O1	163.42 (7)	C8—C7—H7	120.6
O3—Zn1—O1	86.10 (7)	C6—C7—H7	120.6
O9—Zn1—O1	84.93 (7)	C7—C8—O1	124.8 (2)
O2—Zn1—O1	72.04 (6)	C7—C8—C9	121.5 (2)
N1—Zn1—O1	73.67 (6)	O1—C8—C9	113.7 (2)
C8—O1—C10	119.01 (18)	N1—C9—C4	122.7 (2)
C8—O1—Zn1	114.78 (14)	N1—C9—C8	118.74 (19)
C10—O1—Zn1	114.98 (13)	C4—C9—C8	118.6 (2)
C11—O2—Zn1	120.10 (14)	O1—C10—C11	105.98 (18)
N3—O3—Zn1	124.92 (15)	O1—C10—H10A	110.5
N4—O6—Zn1	123.50 (16)	C11—C10—H10A	110.5
Zn1—O9—H9A	109.5	O1—C10—H10B	110.5
Zn1—O9—H9B	135.6	C11—C10—H10B	110.5
H9A—O9—H9B	114.8	H10A—C10—H10B	108.7
C1—N1—C9	118.3 (2)	O2—C11—N2	124.8 (2)
C1—N1—Zn1	123.78 (16)	O2—C11—C10	121.6 (2)
C9—N1—Zn1	117.69 (14)	N2—C11—C10	113.54 (19)
C11—N2—C12	129.5 (2)	C17—C12—C13	120.1 (2)
C11—N2—H2	115.3	C17—C12—N2	123.6 (2)
C12—N2—H2	115.3	C13—C12—N2	116.3 (2)
O5—N3—O4	122.1 (2)	C12—C13—C14	120.1 (3)
O5—N3—O3	120.4 (2)	C12—C13—H13	120.0
O4—N3—O3	117.5 (2)	C14—C13—H13	120.0
O7—N4—O8	123.7 (2)	C15—C14—C13	120.1 (3)
O7—N4—O6	119.6 (2)	C15—C14—H14	120.0
O8—N4—O6	116.7 (2)	C13—C14—H14	120.0
N1—C1—C2	122.5 (3)	C14—C15—C16	119.7 (3)
N1—C1—H1	118.7	C14—C15—H15	120.1
C2—C1—H1	118.7	C16—C15—H15	120.1
C3—C2—C1	119.7 (3)	C15—C16—C17	121.1 (3)
C3—C2—H2A	120.2	C15—C16—H16	119.5
C1—C2—H2A	120.2	C17—C16—H16	119.5
C2—C3—C4	120.1 (2)	C16—C17—C12	119.0 (3)
C2—C3—H3	119.9	C16—C17—H17	120.5
C4—C3—H3	119.9	C12—C17—H17	120.5

### Hydrogen-bond geometry (Å, °)

**Table:**

*D*—H···*A*	*D*—H	H···*A*	*D*···*A*	*D*—H···*A*
O9—H9A···O6^i^	0.82	1.99	2.803 (3)	173
O9—H9B···O4^ii^	0.88	1.97	2.797 (3)	155
N2—H2···O8^iii^	0.86	2.07	2.869 (3)	155

Symmetry codes: (i) −*x*+1, −*y*+2, −*z*; (ii) *x*+1, *y*, *z*; (iii) *x*, *y*−1, *z*.
